# A Bibliometric Analysis of COVID-19 Scientific Literature From the English-Speaking Caribbean

**DOI:** 10.7759/cureus.30958

**Published:** 2022-11-01

**Authors:** Eeshika Chatterjee, Talha Khan, Laura S Renzi, Earlan J Charles, Kesava Mandalaneni

**Affiliations:** 1 Neuroscience, St. George's University School of Medicine, St. George's, GRD

**Keywords:** covid-19, global health diplomacy, trinidad and tobago, barbados, jamaica, vaccine hesitancy, grenada, the university of the west indies, west indies, caribbean

## Abstract

The COVID-19 pandemic has led to a global crisis and has affected the Caribbean islands, leading to significant health and socioeconomic consequences in this region. Efforts to mitigate the burden of this disease have led to an accelerated amount of research in the English-speaking Caribbean (ESC). This bibliometric analysis aimed to evaluate the COVID-19-related scientific literature from the ESC nations. A total of 175 articles were included and analyzed from an initial PubMed search (n = 638) for COVID-19-related scientific literature from the ESC nations published between January 1, 2020, and June 30, 2022. Microsoft Excel 2016 (Microsoft Corporation, Redmond, Washington) and the VOSviewer (version 1.6.18) were used to characterize countries, authorship, journals, affiliations, and keywords of the COVID-19-related articles. Trinidad and Tobago (38%), Jamaica (22%), Barbados (20%), and Grenada (15%) contributed to the greatest number of publications. The University of the West Indies (UWI) campuses in Trinidad and Tobago, Jamaica and Barbados, and St. George's University in Grenada were the most prolific institutions. Srikanth Umakanthan from the UWI was the most prolific author. *Cureus, SN Comprehensive Clinical Medicine*, and *Frontiers in Public Health* were the first three most productive journals; 59% of the 175 articles had either the first or last author affiliated with an institution in the ESC, and 19% of the articles were country-focused: Trinidad and Tobago (16/175), Jamaica (9/175), Barbados (5/175), and Antigua and Barbuda (2/175). Among the top themes of research, 27% were outbreak response and rearrangements, epidemiological studies (23%), clinical management (23%), and medical education (13%). Over the last two years, an interest stimulated by the pandemic has expanded the research in ESC countries. However, gaps in the knowledge exist, especially in the epidemiology of COVID-19 complications in the sub-populations of chronic non-communicable diseases, post-COVID syndrome, and the long-COVID syndrome in the region. Hence, there is enormous scope for more research across the region.

## Introduction and background

In late 2019, a novel beta coronavirus was reported as causing severe acute respiratory syndrome in Wuhan, People's Republic of China, and was named a new coronavirus (2019-nCoV) [1]. In 2020, the virus started spreading to other parts of the world, including Caribbean nations [1,2]. Since its declaration as a global pandemic on March 11, 2020, by the World Health Organization (WHO), COVID-19 has exposed numerous inequalities on an individual and systemic level. While the pandemic has led to a global crisis, it has also affected the Caribbean islands, their people, the health infrastructure, and its heavily tourism-dependent economies [3]. Efforts to mitigate the burden of this disease have led to an accelerated amount of research to identify the restructuring that must be accomplished to operate under the new "normal" COVID-19 conditions [4]. Research with local perspectives aimed at producing region-specific data is essential to address the unique and specific needs of a region. Considering COVID-19 has had a significant impact on the ESC nations, this study aimed to conduct a bibliometric review of the COVID-19 research output and trends from the ESC nations. Bibliometric reviews have been used to analyze written publications quantitatively [5]. It includes classifying the publications in a subject area using statistical tools and analyzing variables like authorship (first vs last vs middle), institutional affiliations, countries, regions, and types of literature, among many others [6]. This study aimed to answer the following research questions using bibliometric analysis: How many COVID-19-related publications have originated from the English-speaking Caribbean (ESC) nations? Which countries are most active in COVID-19-related research? Which institutions are most active in this research? What kind of research has been performed? Who performed COVID-19-related research in the ESC nations?

## Review

Methods

Only the PubMed database of the National Center for Biotechnology Information (NCBI) was used to find the publications related to this study. Detailed search strategy is specified with the keywords used:(((((((((((((("caribbean"[All Fields]) OR ("west indies"[All Fields])) AND ("covid 19"[All Fields])) AND ("jamaica"[All Fields])) OR (((("caribbean"[All Fields]) OR ("west indies"[All Fields])) AND ("covid 19"[All Fields])) AND ("grenada"[All Fields]))) OR (("trinidad and tobago"[All Fields]) AND ((("caribbean"[All Fields]) OR ("west indies"[All Fields])) AND ("covid 19"[All Fields])))) OR (((("caribbean"[All Fields]) OR ("west indies"[All Fields])) AND ("covid 19"[All Fields])) AND ("anguilla"[All Fields]))) OR (((("caribbean"[All Fields]) OR ("west indies"[All Fields])) AND ("covid 19"[All Fields])) AND ("barbados"[All Fields]))) OR (((("caribbean"[All Fields]) OR ("west indies"[All Fields])) AND ("covid 19"[All Fields])) AND ("guyana"[All Fields]))) OR (("antigua and barbuda"[All Fields]) AND ((("caribbean"[All Fields]) OR ("west indies"[All Fields])) AND ("covid 19"[All Fields])))) OR ((St.Kitts and Nevis) AND ((("caribbean"[All Fields]) OR ("west indies"[All Fields])) AND ("covid 19"[All Fields])))) OR (("belize"[All Fields]) AND ((("caribbean"[All Fields]) OR ("west indies"[All Fields])) AND ("covid 19"[All Fields])))) OR (("dominica"[All Fields]) AND ((("caribbean"[All Fields]) OR ("west indies"[All Fields])) AND ("covid 19"[All Fields])))) OR ((St.Lucia) AND ((("caribbean"[All Fields]) OR ("west indies"[All Fields])) AND ("covid 19"[All Fields])))) OR (((St.Vincent and the Grenadines) AND ((("caribbean"[All Fields]) OR ("west indies"[All Fields])) AND ("covid 19"[All Fields]))) AND (St)) “studying”[All Fields) AND (“internet”[MeSH Terms] OR “internet”[All Fields] OR “internet s”[All Fields] OR “internets”[All Fields])).

Inclusion Criteria

The study included only articles in English from ESC nations that focused on COVID-19, in which at least one researcher represented an ESC Institution, with no restrictions on the type of articles, and articles published between January 1, 2020, and June 30, 2022.

Exclusion Criteria

Non-English publications published before and after the above dates that were unrelated to COVID-19 were excluded from the study.

**Figure 1:**
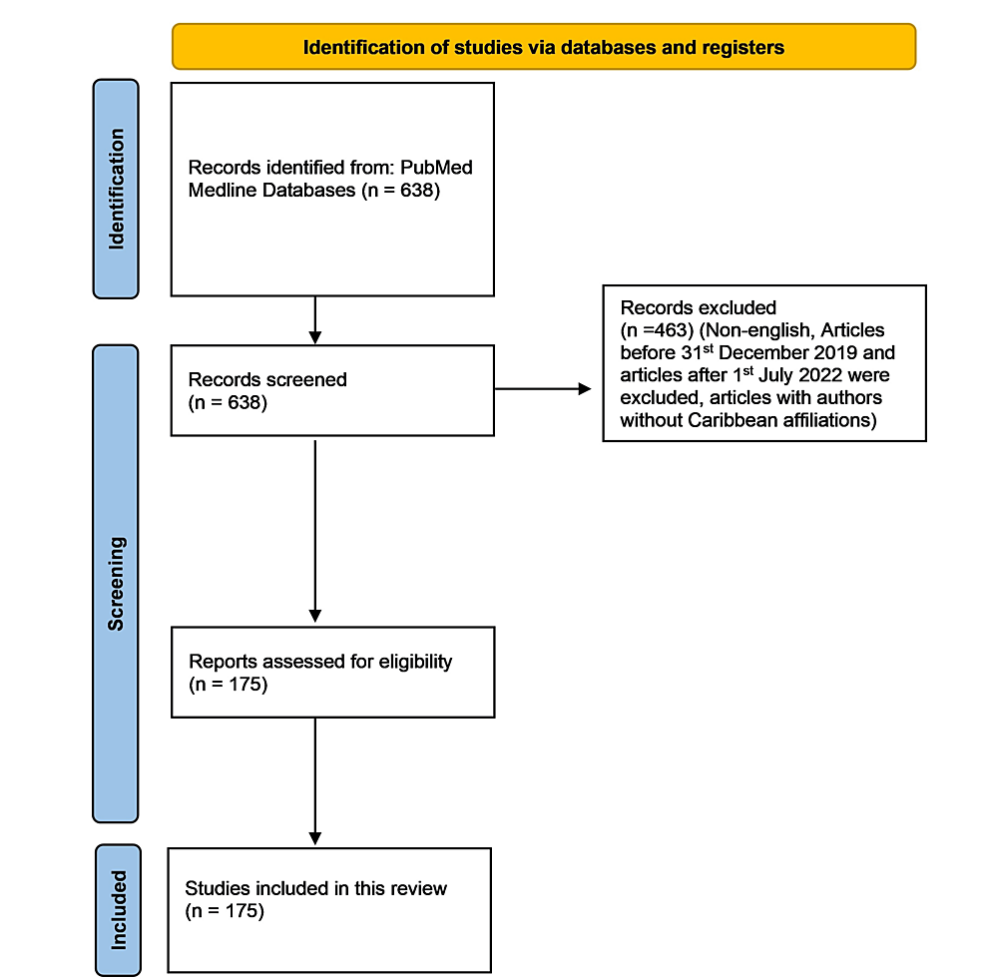
PRISMA flowchart of the systematic review for Caribbean COVID-19 scientific literature The screening performed for this literature review follows guidelines in the PRISMA statement [7]. Image credit: This figure was created by the corresponding author (KM) using a PRISMA template [7]. PRISMA: Preferred Reporting Items for Systematic Reviews and Meta-Analyses.

Four reviewers (TK, EC, EJC, and KM) independently performed article screening. In case of conflict, it was resolved with discussion among the reviewers. Full articles were obtained whenever possible, and references were reviewed for appropriate citations. The inclusion and exclusion process is illustrated in Figure 1. The following elements were analyzed using Microsoft Excel (Microsoft Corporation, Redmond, Washington) and VOSviewer: research themes, number of publications per country, institutional affiliations of the authors in publications, journals in which COVID-19 research from the Caribbean was published, the first author (Institutional affiliation, country, journal frequency, and frequent first authors), and the last author (institutional affiliation, country, journal frequency, and frequent last authors). A modified version [3] of the WHO-coordinated global research roadmap classification [4] of COVID-19 research was used to identify research themes.

Results and analysis

**Figure 2:**
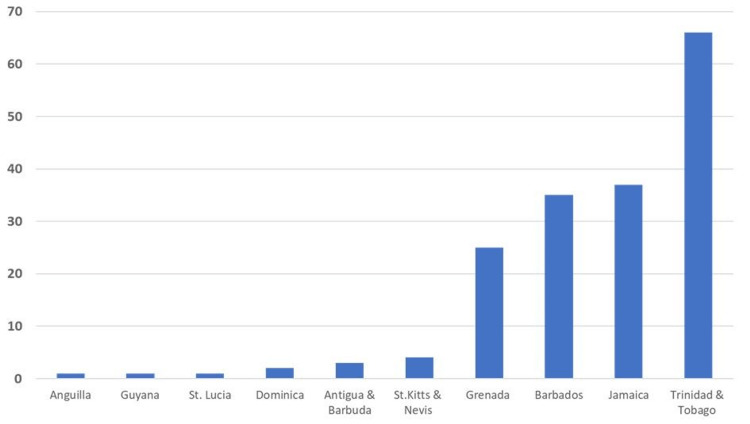
Total number of publications per country Image credit: This figure was created by the corresponding author (KM).

A total of 175 publications from 123 sources were included in the study. From the initial search results (n = 638), 463 articles were excluded because they did not meet the inclusion criteria. Of the 175 publications, most originated from Trinidad and Tobago (38%), Jamaica (22%), Barbados (20%), and Grenada (15%). Smaller countries like Anguilla, St. Lucia, Dominica, Antigua and Barbuda (3%), and St. Kitts and Nevis (2%) have also contributed to the COVID-19 scientific literature. Nineteen percent of the articles were country-focused (the research setting was one of the ESC countries) as led by Trinidad and Tobago (16/175), Jamaica (9/175), Barbados (5/175), and Antigua and Barbuda (2). About 59% of COVID-19-related articles from the region had first and/or last authors affiliated with a Caribbean institution. Ten ESC countries had COVID-19-related research output. VOSviewer software [8] was used to visualize and analyze the tags of published articles (Figure 2).

Analysis of Research Themes

**Figure 3:**
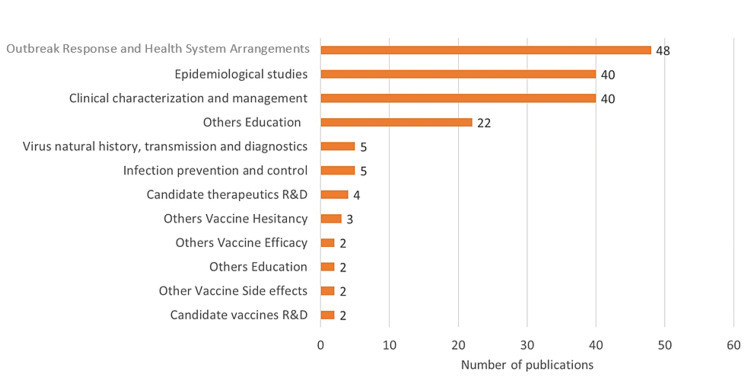
Results of thematic areas of COVID-19 research according to the modified WHO global research roadmap classification of COVID-19 research R&D: Research and development; WHO: World Health Organization. Image credit: This figure was created by the corresponding author (KM).

Among the WHO themes of research, 27% were outbreak response and rearrangements, epidemiological studies (23%), clinical management (23%), and medical education (13%). Outbreak response themes included a recurring subtheme of global health diplomacy's role in overcoming inequity in vaccine rollout in the face of the pandemic. Like many parts of the lower- and middle-income countries (LMIC), there was no mention of or presence of any vaccine-associated randomized control trials (RCTs) [9]. Figure 3 represents the classification of COVID-19 global research as prescribed by WHO.

Number of Publications Per Country

Of the 175 publications, most originated from Trinidad and Tobago (38%), Jamaica (22%), Barbados (20%), and Grenada (15%). In addition, 19% of the articles were country-focused in which the research setting was one of the ESC countries. They included research output as led by Trinidad and Tobago (16/175), Jamaica (9/175), Barbados (5/175), and Antigua and Barbuda (2/175).

Analysis of Journals 

**Figure 4:**
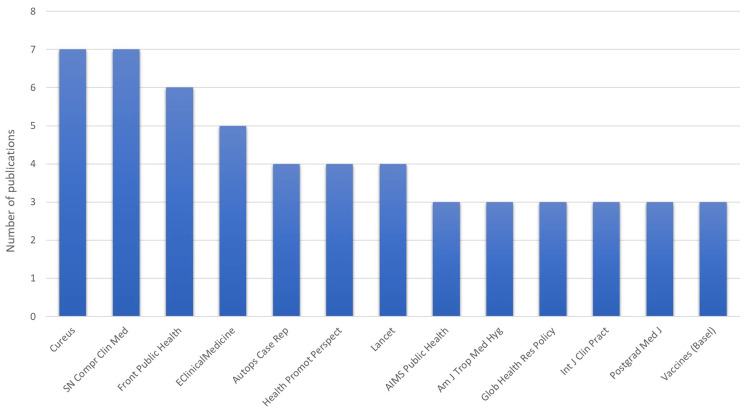
Journals in which COVID-19 research from the Caribbean was published SN Compr Clin Med: SN Comprehensive Clinical Medicine; Front Public Health: Frontiers in Public Health; Autops Case Rep: Autopsy and Case Reports; Health Promot Perspect: Health Promotion Perspectives; Glob Health Res Policy: Global Health Research and Policy; Int J Clin Pract: International Journal of Clinical Practice; Postgrad Med J: Postgraduate Medical Journal; Am J Trop Med Hyg: The American Journal of Tropical Medicine and Hygiene. Image credit: This figure was created by the corresponding author (KM).

Figure 4 represents journals in which COVID-19 research from the Caribbean was published. They include journals like *Frontiers in Public Health*, *Lancet*, *Nature*, and *Cureus.*

*Author Analysis (First Author, Last Author* *, and Institutional Affiliations)*

**Figure 5:**
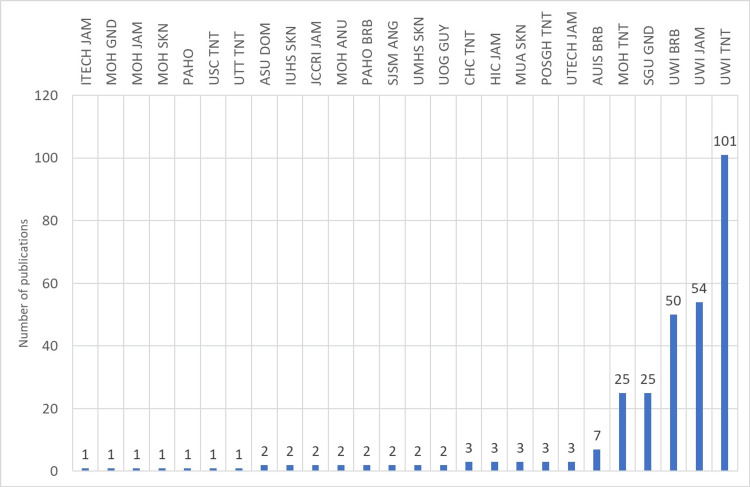
Affiliations of the Caribbean authors MOH: Ministry of Health; AUIS: American University of Integrative sciences; UWI: University of the West Indies; PAHO: Pan American Health Organization; UOG: University of Guyana; HIC: Heart Institute of the Caribbean; MUA: Medical University of the Americas; UMHS: University Medical and Health Sciences; CHC: Caribbean Heart Care; USC: University of the Southern Caribbean; POSGH: Port of Spain General Hospital; JCCRI: Jamaica Cancer Care and Research Institute; SJSM: St. James University School of Medicine; SGU: St. George’s University; PAHO: Pan American Health Organization; UTT: University of Trinidad and Tobago; ASU: All Saint’s University School of Medicine; ANU: Antigua and Barbuda; ANG: Anguilla; BRB: Barbados; GUY: Guyana; GND: Grenada; SKN: St. Kitts and Nevis; SLU: St. Lucia; TNT: Trinidad and Tobago; JAM: Jamaica. Image credit: This figure was created by the corresponding author (KM).

**Figure 6:**
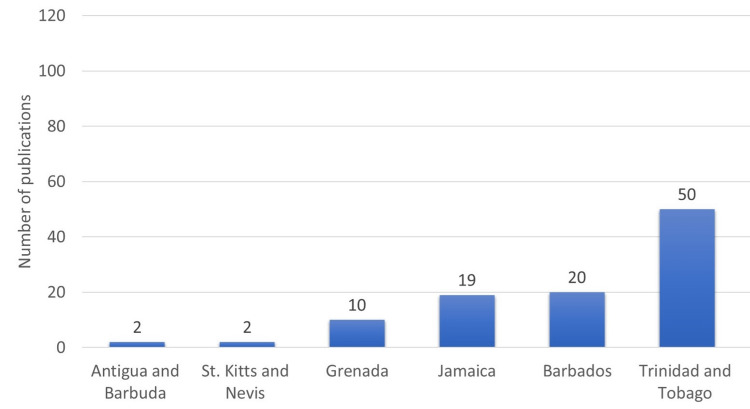
First author (n) by country Image credit: This figure was created by the corresponding author (KM).

**Figure 7:**
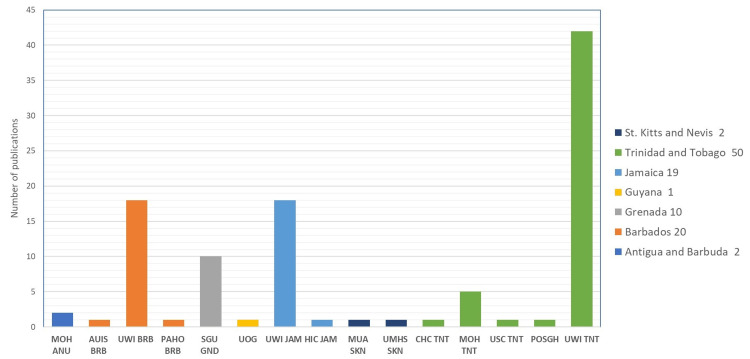
Institutional affiliations of the first authors MOH: Ministry of Health; AUIS: American University of Integrative Sciences; UWI: University of the West Indies; PAHO: Pan American Health Organization; UOG: University of Guyana; HIC: Heart Institute of the Caribbean; MUA: Medical University of the Americas; UMHS: University Medical and Health Sciences; CHC: Caribbean Heart Care; USC: University of the Southern Caribbean; POSGH: Port of Spain General Hospital; JCCRI: Jamaica Cancer Care and Research Institute; SJSM: St. James University School of Medicine; SGU: St. George’s University; PAHO: Pan American Health Organization; UTT: University of Trinidad and Tobago; ASU: All Saint’s University School of Medicine; ANU: Antigua and Barbuda; ANG: Anguilla; BRB: Barbados; GUY: Guyana; GND: Grenada; SKN: St. Kitts and Nevis; SLU: St. Lucia; TNT: Trinidad and Tobago; JAM: Jamaica. Image credit: This figure was created by the corresponding author (KM).

**Figure 8:**
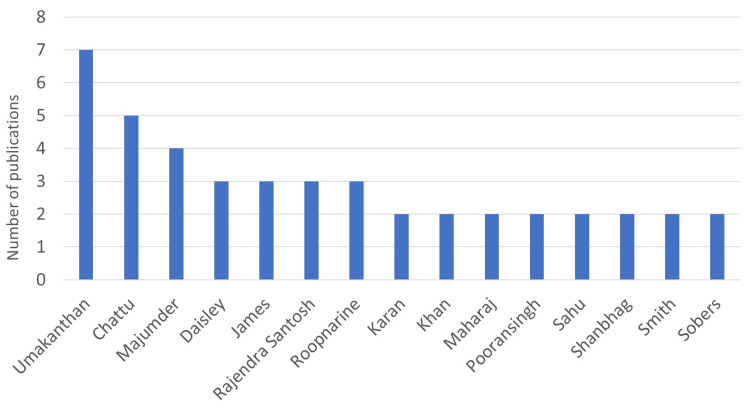
Frequent first authors Image credit: This figure was created by the corresponding author (KM).

**Figure 9:**
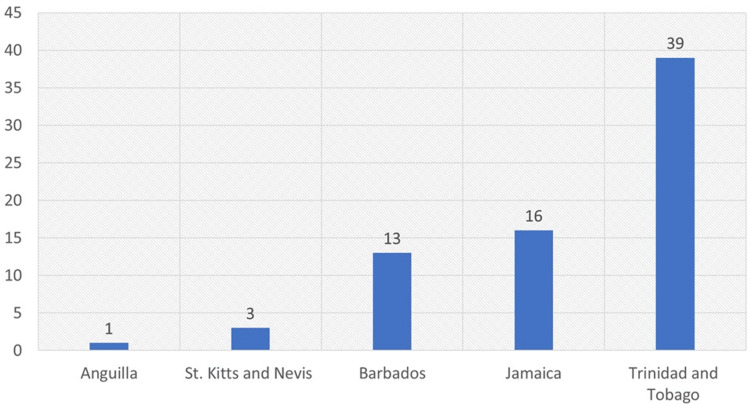
Last author (n) by country Image credit: This figure was created by the corresponding author (KM).

**Figure 10:**
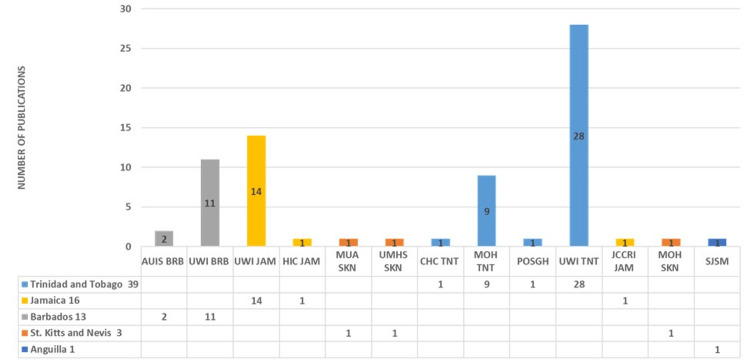
Institutional affiliations of the last authors MOH: Ministry of Health; AUIS: American University of Integrative Sciences; UWI: University of the West Indies; PAHO: Pan American Health Organization; UOG: University of Guyana; HIC: Heart Institute of the Caribbean; MUA: Medical University of the Americas; UMHS: University Medical and Health Sciences; CHC: Caribbean Heart Care; USC: University of the Southern Caribbean; POSGH: Port of Spain General Hospital; JCCRI: Jamaica Cancer Care and Research Institute; SJSM: St. James University School of Medicine; SGU: St. George’s University; PAHO: Pan American Health Organization; UTT: University of Trinidad and Tobago; ASU: All Saint’s University School of Medicine; ANU: Antigua and Barbuda; ANG: Anguilla; BRB: Barbados; GUY: Guyana; GND: Grenada; SKN: St. Kitts and Nevis; SLU: St. Lucia; TNT: Trinidad and Tobago; JAM: Jamaica. Image credit: This figure was created by the corresponding author (KM).

**Figure 11:**
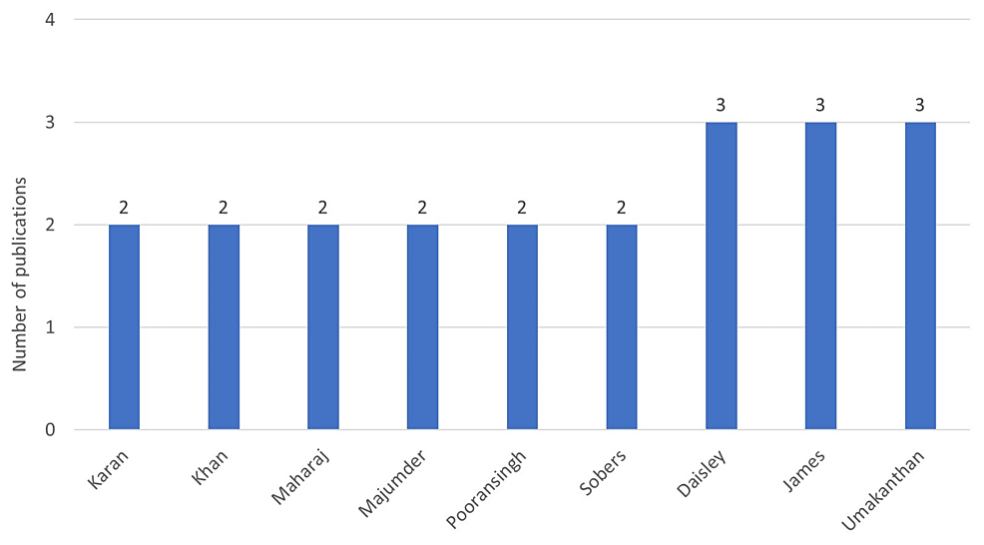
Frequent last authors Image credit: This figure was created by the corresponding author (KM).

**Figure 12:**
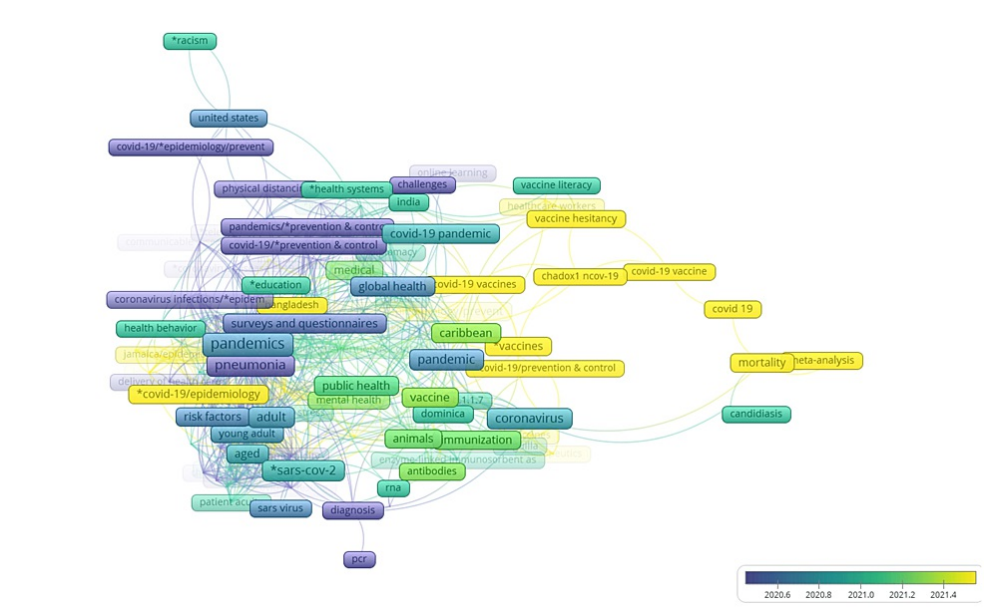
VOSviewer overlay visualization of tag analysis of the published articles, observe the transition (of tags) from 2020 to 2022 Image credit: This figure was created by the corresponding author (KM) using the software VOSviewer based on the tags of the articles retrieved in the results.

An analysis of first author affiliations shows that most of the first authors are from Trinidad and Tobago (48%), most notably from the University of the West Indies (UWI), followed by UWI campuses in Barbados (20%) and Jamaica (19%). St. Georges University (SGU) in Grenada (10%) had the most first author articles among the smaller island nations that do not have a campus of the UWI. The first authors from the Caribbean have published their research articles in journals like *Frontiers in Public Health, BMJ, and Lancet*. Country-wise, frequent first authors are led by Trinidad and Tobago (50), most notably from the UWI, followed by UWI campuses in Barbados (19) and Jamaica (20), and St. George's University in Grenada (10). A numerical analysis of the last author's affiliation and countries shows the same trend as the first authors, with the UWI in Trinidad in the lead, followed by its campuses in Jamaica and Barbados. Figures 5-12 represent the authors' institutional affiliations, first author by country, institutional affiliations of the first authors, frequent first authors, last author by country, institutional affiliations of the last authors, and frequent last authors. The top two contributing authors for COVID-19-related research from the ESC were Srikanth Umakanthan and Vijay Kumar Chattu, both from the UWI (Trinidad and Tobago).

Discussion

Researchers from ESC have published articles on wide-ranging topics. The scramble for adaptation and preparedness in the face of a crisis echoed in the early 2020 articles has evolved in 2022 into articles addressing vaccine side effects and other refined topics.

2020: First Cases, Waves, Lockdowns, Zoom Education, and Unchartered Seas

COVID-19 has necessitated and expedited the usage of the telemedicine platform for care delivery to patients. The earliest published articles from the Caribbean included the use of telemedicine across specialties. Researchers collaborated internationally and were part of a consortium under the aegis of the WHO, which has explored the possibilities of telemedicine across specialties like cardiology, non-acute care, palliative care, mental health, and neurology [10,11]. The impact/crisis and preparedness articles [12,13] have featured mainly in the earlier months of COVID-19. One impact study included not only commentaries about the disruption of neonatal services [14], but these Barbadian researchers also proposed protocols and published them for use by the wider clinical community dealing with neonatal care in the face of COVID-19. The cardiac surgery department in Trinidad and Tobago developed protocols to ensure the safe delivery of care to patients, which included maintaining a COVID-19 cold site, social isolation of patients for one to two weeks before surgery, and other measures as they rearranged its department to cater to emergency and elective cardiac surgery in the face of COVID-19 [15].

Cancer is among the major chronic non-communicable diseases within the ESC. Therefore, it is not surprising to see cancer and care delivery as a recurring theme in these publications in 2020 [16,17]. The main focus was to cater to care delivery in COVID-19 times and was also prominently featured in *The Lancet* oncology [16]. The region is home not only to the University of the West Indies and its three campuses of medicine in Jamaica, Barbados, Trinidad, and Tobago but also to many offshore medical universities dotted across the Caribbean. Therefore, it has not been surprising to see studies related to the disruption of medical education and emerging innovation in the delivery of the curriculum [18]. Cadaveric dissection, small group learning, and didactic teaching have all evolved into a setting that has scarcely been used till 2020. Medical education has adapted new teaching methodologies using technology [19]. When COVID-19 treatment was still uncharted territory, there were concerns about increased antimicrobial resistivity (AMR) and polypharmacy. Insightful researchers from Barbados addressed the topic of AMR in light of an era when there could have been possible indiscriminate usage of antibiotics [20,21]. These articles have suggested a holistic and multisector approach to address AMR, its rising threat, and its possible impact during the pandemic [20].

2021: Vaccine Rollout and Vaccine Hesitancy

2021 ushered in a new chapter in the pandemic with the advent of vaccines against COVID-19. Much of the publications from the Caribbean revolved around vaccines. Researchers also have written about how global health diplomacy can be harnessed to counter the dominance of profit-oriented businesses and build capacity in the face of COVID-19 toward improved outcomes and regional economic recovery. Health policy researchers constantly stressed the role of global health diplomacy in bridging vaccine gaps and prioritizing vaccine equity in the global health agenda in the fight against COVID-19 [22-24] and highlighted how the resolution of a pandemic could not happen just by one or a few healthcare systems but by collaboration and global solidarity. They observed that at the end of 18 months of the pandemic, only five countries had developed a vaccine. Hence, the only way forward for the collective recovery of people around the world and the global economy is with health diplomacy. The foresight and ideas of Caribbean researchers in global health policy are evident if one reads these articles chronologically. Caribbean researchers advocated the importance of health diplomacy to address vaccine acquisition and vaccine equity in many (LMIC) countries and thus propel economic recovery [25]. Vaccine rollout logistics also included battling misinformation about vaccine hesitancy in the general population and healthcare providers [26]. Comments made by a famous singer, Ms. Nikki Minaj, about the possibility of impotence due to vaccines have created a furor and undoubtedly added to the travails of the region's already fragile vaccination drive [27,28].

The trend of articles about medical education continued within the region [29]. The articles are now more refined and explored topics such as whole slide imaging as a method to teach the residents in pathology [30]. Not only education but assessments were also conducted virtually for the postgraduate trainees at the UWI as there was an urgent and pressing need to graduate well-trained specialists in time to battle the COVID-19 pandemic [31]. Roopnarine et al. suggested the importance of the conceptual framework of one health (OH) in medical, veterinary, and public health programs [32,33]. They opined that COVID-19 has proven that such an education in the curriculum is vital in preparing students for current and future threats to global health. COVID-19 has affected dental surgeons professionally, financially, and mentally. Aerosol generation during dental procedures is deemed to have a very high risk for COVID-19 transmission. Therefore, dental surgeons were considered at a very high risk of exposure to this virus. Researchers have detailed many aspects of dentistry and the travails of practicing dentistry during the COVID-19 pandemic [34]. The Caribbean dental researchers [35] were also at the forefront of addressing the pathogenesis, clinical features, diagnosis, and treatment of oral mycoses in the face of COVID-19. They also specifically addressed the emerging threat of mucormycosis [36] in the face of chronic steroid use in COVID-19 treatment. Stay-at-home orders to curb the pandemic have had several ramifications that have become apparent in the earlier periods of the pandemic. Intimate partner violence, also referred to as domestic violence, was addressed notably in this article's commentary [37]. The clinician researchers suggested implementing WHO's LIVES (Listen, Inquire, Validate, Enhance safety, and provide Support) framework for inquiring and responding to a disclosure of domestic in telehealth consultations [37].

2022: Refined Literature and Hopefully an End of the Pandemic

The year 2022 saw the spread of a new variant, B.1.1.529 (Omicron) [38], across the world, including the Caribbean. However, by this time, many of the Caribbean countries had rolled out their vaccination programs with the vaccines procured by various means, most notably via the COVAX initiative (COVID-19 Vaccines Global Access) [39] and through donations and diplomacy from the Republic of India, the People’s Republic of China, the USA, the Russian Federation, and the European Union. The repertoire of vaccines included Oxford AstraZeneca, Pfizer, Sinopharm, Moderna, and Sputnik. Almost 25% of research articles from the ESC in the first half of 2022 focused on vaccines. The vaccine themes included side effects [40] IgG responses to vaccines [41], booster doses [42], and vaccine hesitancy.

Randomized control trials (RCT) are the gold standard for determining causal links and most accurately measure the efficacy of novel interventions and treatments. Unfortunately, like many parts of the LMICs, there was no mention of or presence of any vaccine-associated RCTs [9]. Vaccine hesitancy in the ESC may have been brought on by this lack/underrepresentation of locally conducted vaccine studies and overreliance on non-Caribbean countries for vaccine usage and COVID-19 prevention data. The Caribbean region predominantly has people of African heritage, and the vaccine hesitancy/distrust may also have roots in the historical atrocities committed against Afro-Americans, for example, in the Tuskegee study [43]. Barbados Diabetes Remission Study-2 is an ongoing clinical trial and singularly stands out as being the only region-specific article related to an ongoing clinical trial [44]. Online medical education, as reflected in the articles in 2022, has streamlined much more, a far cry from the trial-and-error scramble for adaptation to a virtual learning environment in 2020. Commentaries included best practices in online teaching [45], having possibly tried and tested many ways. An interesting article featured senior surgeons successfully distance mentoring postgraduate surgical (PGY4/5) residents by virtual technology in 22 laparotomy-required trauma cases [46].

Comment About Case Reports 2020-2022

The intellectual curiosity of the Caribbean clinician researchers is evident if one were to explore the articles related to comorbidity, case reports, and clinical findings in the published articles from the region. They have added unique and region-specific clinical findings to the worldwide COVID-19 scientific literature. Most notably, such region-specific case reports and clinical findings came from Barbados, Jamaica, and Trinidad and Tobago. Some of the notable articles include atrial arrhythmia findings and its treatment in Trinidad [47], cardiac findings in COVID-19 infections [48], usage of a short course of prednisone in COVID-19 patients, and how it helped in preventing worsening of shortness of breath in early COVID respiratory failure [49]. Prominent among literature with autopsy findings is the vivid description of pathologies of alveolar microcirculation at pericytes [50,51] with a high concentration of angiotensin-converting enzyme 2 (ACE2) receptors, which are believed to be the primary site of action of SARS-CoV-2.

Limitations

A significant limitation of this study is the inclusion of only PubMed-listed English articles produced in ESC nations. Institutional affiliation as a sign of the author's nationality poses a limitation to this analysis. Since authors with non-Caribbean nationalities working in institutions within ESC were categorized as the Caribbean, this analysis may suggest an overrepresentation of Caribbean authors. On the other hand, the converse may also be true, in which there may be a significant underrepresentation of Caribbean authors affiliated with non-Caribbean institutions.

## Conclusions

Since the beginning of the COVID-19 pandemic in 2020, scientists and public health experts throughout the ESC nations have responded to the urgent need for COVID-19-related research efforts. This article guides researchers with COVID-19 publication trends in the ESC nations. Research in the region evolved from an initial focus on adaptation, preparedness, and comorbidities to vaccines and vaccine-related issues. Most research originated from Trinidad and Tobago (38%), Jamaica (22%), Barbados (20%), and Grenada (15%). Researchers from the ESC region were not only able to identify region-specific COVID-19-related obstacles but also made significant contributions to international literature concerning the global pandemic. Continued research from the ESC nations, including smaller countries, is essential for the ongoing management of the pandemic and chronic non-communicable diseases.

## References

[1] Zhou F, Yu T, Du R (2020). Clinical course and risk factors for mortality of adult inpatients with COVID-19 in Wuhan, China: a retrospective cohort study. Lancet.

[2] Guan WJ, Ni ZY, Hu Y (2020). Clinical characteristics of coronavirus disease 2019 in China. N Engl J Med.

[3] Mansilla C, Herrera CA, Boeira L (2022). Characterising COVID-19 empirical research production in Latin America and the Caribbean: a scoping review. PLoS One.

[4] (2022). A coordinated global research roadmap. https://www.who.int/publications/m/item/a-coordinated-global-research-roadmap.

[5] Ellegaard O, Wallin JA (2015). The bibliometric analysis of scholarly production: how great is the impact?. Scientometrics.

[6] Ma Y, Dong M, Zhou K, Mita C, Liu J, Wayne PM (2016). Publication trends in acupuncture research: a 20-year bibliometric analysis based on PubMed. PLoS One.

[7] Liberati A, Altman DG, Tetzlaff J (2009). The PRISMA statement for reporting systematic reviews and meta-analyses of studies that evaluate healthcare interventions: explanation and elaboration. BMJ.

[8] (2022). Visualizing scientific landscapes. https://www.vosviewer.com/.

[9] Guleid FH, Oyando R, Kabia E, Mumbi A, Akech S, Barasa E (2021). A bibliometric analysis of COVID-19 research in Africa. BMJ Glob Health.

[10] Bhaskar S, Bradley S, Chattu VK (2020). Telemedicine as the New Outpatient Clinic Gone Digital: Position Paper From the Pandemic Health System REsilience PROGRAM (REPROGRAM) International Consortium (Part 2). Front Public Health.

[11] Bhaskar S, Bradley S, Sakhamuri S (2020). Designing futuristic telemedicine using artificial intelligence and robotics in the COVID-19 era. Front Public Health.

[12] Andrus JK, Evans-Gilbert T, Santos JI (2020). Perspectives on battling COVID-19 in countries of Latin America and the Caribbean. Am J Trop Med Hyg.

[13] King A, Andrus JK, Figueroa JP (2020). Financial crisis at PAHO in the time of COVID-19: a call for action. Lancet.

[14] Kalyanasundaram S, Krishnamurthy K, Sridhar A, Narayanan VK, Rajendra Santosh AB, Rahman S (2020). Novel corona virus pandemic and neonatal care: it’s too early to speculate on impact!. SN Compr Clin Med.

[15] Ramsingh RA, Duval JL, Rahaman NC, Rampersad RD, Angelini GD, Teodori G (2020). Adult cardiac surgery in Trinidad and tobago during the COVID-19 pandemic: lessons from a developing country. J Card Surg.

[16] Andall-Brereton G, Bromfield B, Smith S, Spence D (2020). Cancer care in the commonwealth Caribbean in COVID times. Lancet Oncol.

[17] Shanbhag NM, Phillip JC, Duncan A (2020). Managing cancer care during the COVID-19 pandemic-experience at a cancer department in a tertiary hospital in Antigua and Barbuda. Pan Afr Med J.

[18] Sahu P (2020). Closure of universities due to coronavirus disease 2019 (COVID-19): impact on education and mental health of students and academic staff. Cureus.

[19] Gaur U, Majumder MA, Sa B, Sarkar S, Williams A, Singh K (2020). Challenges and opportunities of preclinical medical education: COVID-19 crisis and beyond. SN Compr Clin Med.

[20] Majumder MA, Rahman S, Cohall D, Bharatha A, Singh K, Haque M, Gittens-St Hilaire M (2020). Antimicrobial stewardship: fighting antimicrobial resistance and protecting global public health. Infect Drug Resist.

[21] Rahman S, Singh K, Dhingra S (2020). The double burden of the COVID-19 pandemic and polypharmacy on geriatric population - public health implications. Ther Clin Risk Manag.

[22] Chattu VK, Adisesh A, Yaya S (2020). Canada's role in strengthening global health security during the COVID-19 pandemic. Glob Health Res Policy.

[23] Chattu VK, Knight WA, Adisesh A (2021). Politics of disease control in Africa and the critical role of global health diplomacy: a systematic review. Health Promot Perspect.

[24] Chattu VK, Singh B, Kaur J, Jakovljevic M (2021). COVID-19 vaccine, trips, and global health diplomacy: India’s role at the WTO platform. Biomed Res Int.

[25] Chattu VK, Pooransingh S, Allahverdipour H (2021). Global health diplomacy at the intersection of trade and health in the COVID-19 era. Health Promot Perspect.

[26] Ashok N, Krishnamurthy K, Singh K, Rahman S, Majumder MA (2021). High COVID-19 vaccine hesitancy among healthcare workers: should such a trend require closer attention by policymakers?. Cureus.

[27] Maharaj SB, Dookeeram D, Franco DY (2021). The Nikki Minaj effect: the impact of social media disinformation on vaccine hesitancy in the Caribbean. J Glob Health.

[28] (2022). Nicki Minaj: Trinidad minister criticises rapper's vaccine tweet. https://www.bbc.com/news/world-latin-america-58581292.

[29] Majumder MA, Gaur U, Singh K (2021). Impact of COVID-19 pandemic on radiology education, training, and practice: a narrative review. World J Radiol.

[30] Evans AJ, Depeiza N, Allen SG, Fraser K, Shirley S, Chetty R (2021). Use of whole slide imaging (WSI) for distance teaching. J Clin Pathol.

[31] Motilal S, Paul-Charles J, Asnani M (2021). 2020 family medicine postgraduate examinations at the university of the west indies: successes and challenges in the time of COVID-19 pandemic. Postgrad Med J.

[32] Roopnarine R, Boeren E, Regan JA (2021). The missing professional perspective: medical, veterinary, and dual degree public health student perceptions of one health. Front Public Health.

[33] Roopnarine R, Regan JA (2021). Faculty perceptions: a qualitative study of the perceived need, opportunities, and challenges of developing “one health-one medicine” in the medical, veterinary, and public health curricula. J Contin Educ Health Prof.

[34] Balkaran R, Bhat M, Marchan S, Smith W (2021). Knowledge, attitude, and practices of dentists in Caribbean countries during the COVID-19 pandemic: a multicenter cross-sectional study. Dent J (Basel).

[35] Rajendra Santosh AB, Muddana K, Bakki SR (2021). Fungal infections of oral cavity: diagnosis, management, and association with COVID-19. SN Compr Clin Med.

[36] Bharatha A, Kandamaran L, Krishnamurthy K (2021). Commentary: “fungal infections of oral cavity: diagnosis, management, and association with COVID-19”. SN Compr Clin Med.

[37] Khan R, David S (2021). A perspective on intimate partner violence since COVID-19. Front Glob Womens Health.

[38] VanBlargan LA, Errico JM, Halfmann PJ (2022). An infectious SARS-CoV-2 B.1.1.529 Omicron virus escapes neutralization by therapeutic monoclonal antibodies. Nat Med.

[39] (2022). Working for global equitable access to COVID-19 vaccines. https://www.who.int/initiatives/act-accelerator/covax.

[40] Fernandes J, Jaggernauth S, Ramnarine V, Mohammed SR, Khan C, Panday A (2022). Neurological conditions following COVID-19 vaccinations: chance or association?. Cureus.

[41] Leys YE, Nwokocha M, Walker JP (2022). SARS-CoV-2 receptor-binding domain IgG response to AstraZeneca AZD1222 COVID-19 vaccination, Jamaica. Am J Trop Med Hyg.

[42] Krause PR, Rees H, Figueroa JP, Swaminathan S, Restrepo AM (2022). Booster vaccines for COVID-19 vaccine breakthrough cases? - Authors' reply. Lancet.

[43] Bajaj SS, Stanford FC (2021). Beyond Tuskegee - vaccine distrust and everyday racism. N Engl J Med.

[44] Quimby KR, Murphy MM, Harewood H (2022). Adaptation of a community-based type-2 diabetes mellitus remission intervention during COVID-19: empowering persons living with diabetes to take control. Implement Sci Commun.

[45] Sahu PK, Dalcik H, Dalcik C, Gupta MM, Chattu VK, Umakanthan S (2022). Best practices for effective implementation of online teaching and learning in medical and health professions education: during COVID-19 and beyond. AIMS Public Health.

[46] Cawich SO, Mencia M, Thomas D, Spence R, Milne D, Naraynsingh V, Barrow S (2022). Trauma surgery via distance mentoring: a model inspired by the 2020 pandemic. Trop Doct.

[47] Seecheran R, Narayansingh R, Giddings S (2020). Atrial arrhythmias in a patient presenting with coronavirus disease-2019 (COVID-19) infection. J Investig Med High Impact Case Rep.

[48] Goha A, Mezue K, Edwards P, Nunura F, Baugh D, Madu E (2020). COVID-19 and the heart: an update for clinicians. Clin Cardiol.

[49] Ventour D, Sieunarine R, Gopaul C (2022). A short course of prednisolone in patients with moderate COVID 19 respiratory failure-stop the progression a case series. J Investig Med High Impact Case Rep.

[50] Daisley H Jr, Rampersad A, Daisley M, Ramdin A, Acco O (2020). Coronavirus 229E with rhinovirus co-infection causing severe acute respiratory distress syndrome with thrombotic microangiopathy and death during Covid-19 pandemic: lessons to be learnt. Autops Case Rep.

[51] Daisley H Jr, Rampersad A, Daisley M, Ramdin A, Acco O, Narinesingh F, Humphrey O (2021). COVID-19: a closer look at the pathology in two autopsied cases. Is the pericyte at the center of the pathological process in COVID-19?. Autops Case Rep.

